# Utilising a simulation platform to understand the effect of domain model assumptions

**DOI:** 10.1007/s11047-014-9428-7

**Published:** 2014-06-04

**Authors:** Kieran Alden, Paul S. Andrews, Henrique Veiga-Fernandes, Jon Timmis, Mark Coles

**Affiliations:** 1 York Computational Immunology Lab, University of York, York, UK; 2 Centre for Immunology and Infection, University of York and Hull York Medical School, York, UK; 3 Department of Computer Science, University of York, York, UK; 4 Instituto de Medicina Molecular, Faculdade de Medicina de Lisboa, Lisbon, Portugal; 5 Department of Electronics, University of York, York, UK

**Keywords:** Model composition, Peyer’s patches, Simulation, Statistical analysis

## Abstract

Computational and mathematical modelling approaches are increasingly being adopted in attempts to further our understanding of complex biological systems. This approach can be subjected to strong criticism as substantial aspects of the biological system being captured are not currently known, meaning assumptions need to be made that could have a critical impact on simulation response. We have utilised the CoSMoS process in the development of an agent-based simulation of the formation of Peyer’s patches (PP), gut-associated lymphoid organs that have a key role in the initiation of adaptive immune responses to infection. Although the use of genetic tools, imaging technologies and ex vivo culture systems has provided significant insight into the cellular components and associated pathways involved in PP development, interesting questions remain that cannot be addressed using these approaches, and as such well justified assumptions have been introduced into our model to counter this. Here we focus not on the development of the model itself, but instead demonstrate how the resultant simulation can be used to assess how these assumptions impact the simulation response. For example, we consider the impact of our assumption that the migration rate of lymphoid tissue cells into the gut remains constant throughout PP development. We demonstrate that an analysis of the assumptions made in the construction of the domain model may either increase confidence in the model as a representation of the biological system it captures, or may suggest areas where further biological experimentation is required.

## Introduction

Despite great advances in laboratory technologies, a large number of interesting biological questions remain that are difficult to address using currently available experimental techniques. The use of mathematical and computational prediction methods that complement traditional studies has become increasingly prevalent: permitting in silico experimentation under which experimental conditions are controlled, and the ethical, financial, and technological constraints associated with laboratory investigations are avoided (Germain et al. 2011; Andrews et al. 2008).

Computational prediction tools, or simulations, can provide an interpretation of underlying biological data upon which the tool is constructed (Guo and Tay 2005). Yet it may be intractable to capture the complete biological system within the simulation, and in the majority of applications the biological system is not fully understood. This necessitates the introduction of biological assumptions into the model, adding a layer of abstraction between the simulator and the real-world system the tool is to represent. Where the tool is used to make predictions that will inform further study, it is vital that the effect these decisions have on the simulation result is fully understood.

The development of secondary lymphoid organs (SLO) is one case where our current understanding of the biology is incomplete, and is of key interest in understanding how the immune system develops. Reductionist experimental approaches have utilised genetic tools, imaging technologies and ex vivo culture systems to explore the cellular and mechanical components involved (Cupedo and Mebius 2003; van de Pavert and Mebius 2010; Randall et al. 2008; Veiga-Fernandes et al. 2007), but these have yet to address all open interesting biological questions. These SLO’s, which include lymph nodes, Peyer’s patches (PP) and the spleen, are strategically located at lymphatic tissue drainage points, ensuring an adaptive immune response to antigens in peripheral tissue is triggered. Specifically, PP trigger adaptive immune responses in the gastrointestinal tract: the largest area of contact between the host and the exterior environment and thus susceptible to exposure to pathogens (Reis and Mucida 2012). Although laboratory investigations have created a generally accepted model of pre-natal organ development, it remains unclear why PP development is highly stochastic. Pre-natal observations suggest that an average of 60 PP develop in the human fetal gut, yet no two observations are identical (Cornes 1965), with large variation in the number, location, and size of the PP. Similar conclusions have been drawn from mouse observations, where around 8–12 PP develop (Alden et al. 2012). The reason for this variation among individuals, and the effect this has on the immune response, is yet to be fully understood.

We have utilised the CoSMoS Process (Andrews et al. 2010) to develop a computational tool that captures the current biological understanding of PP development (Alden et al. 2012; Patel et al. 2012). The CoSMoS process encourages collaboration between the researcher implementing the simulation and experts in the studied biological system, and proposes a set of activities that lead to the production of a series of models. Collaboration is key in the construction of the first model, the *domain* model, which acts as a specification of the biological system being modelled. As we have noted, it is intractable to capture the complete biological system under study (both the ‘*known*’ and the ‘*unknown*’), and thus assumptions are introduced at this point. Collaboration with an expert in this particular biological field ensures that these assumptions are discussed and agreed before thought turns to tool implementation. Once the biological model is agreed, a *platform* model is generated that specifies how this biological information will be implemented as a computer simulation, noting any simplifications that need to be made. From this, the *simulation platform* is developed, and routines generated for understanding how simulation results can be interpreted in terms of the real-world system the tool represents (the *results* model). The models generated in the development of our PP organogenesis simulation have been made freely available alongside the resultant simulation platform (Alden et al. 2012).

The aim of this paper is not to discuss the development of the simulator itself, but to examine the influence of decisions that are made in the first stage: the construction of the *domain* model. Although we were fortunate that some biological data was available when the PP organogenesis simulation was constructed, key assumptions had to be made that describe the migration of particular cell types into the developing gastrointestinal tract. From these assumptions we developed our *platform* model, and subsequently our *simulation platform*, and have published key results that our tool has generated (Patel et al. 2012; Alden et al. 2012). Whereas it is becoming increasingly prevalent to see researchers utilising sensitivity analysis techniques to explore simulation platform parameters where a value is unknown (Read 2011; Marino et al. 2008), it is rare to find an investigation of the effect that necessary biological assumptions have on the simulation response. These may have a critical impact when determining what a simulation result means in terms of the real-world system.

Below we briefly introduce our available PP organogenesis simulation, and discuss two key cell migration assumptions that we have introduced in the domain model. We then state our methodology for exploring the effect these assumptions have on simulation behaviour, and subsequently show results obtained using this strategy. Finally we discuss the impact assumptions may have on conclusions drawn through simulation, and note the importance of making biological assumption decisions available alongside a computational prediction tool.

## PP organogenesis simulation

As noted above, full descriptions of our *domain*, *platform*, *simulation platform*, and *results* models are available elsewhere (Alden et al. 2012), yet it is important for the context of this paper that we briefly introduce the concepts behind the simulator here. However the description given below is high-level and avoids the complete biological detail that can be found in our published work.

**Fig. 1:**
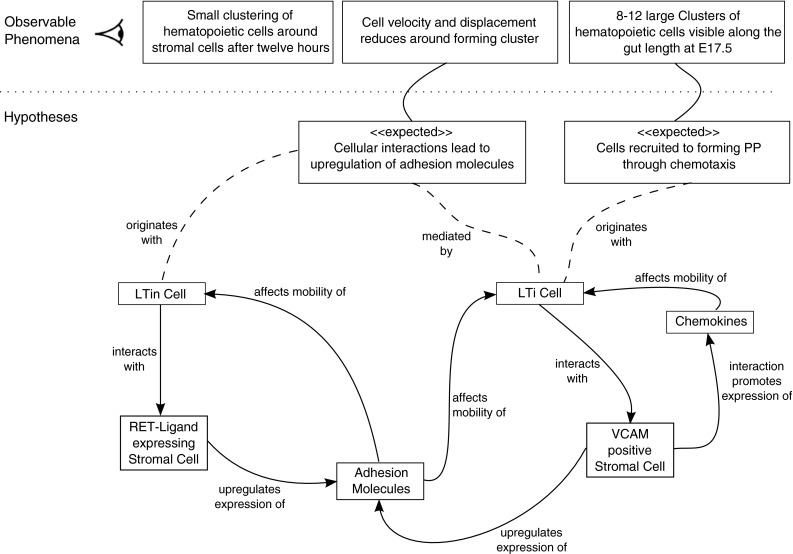
Expected behaviour diagram, detailing the phenomena observed in PP development, the domain being modelled in our simulation. These observations (*above dotted line*) emerge from interations between biological components (*below*). This diagram details the interactions that are currently thought to be responsible for each observation

Figure 1 details the biological phenomenon observed in the development of PP in pre-natal mice, along with current hypotheses for the cause of each observation. Three cell types can be identified from the figure: LTin, or lymphoid tissue initiator cell; LTi, or lymphoid tissue inducer cell; and LTo, or lymphoid tissue organiser cell. Migration of LTin and LTi cells into the developing gut can be detected from embryonic day 14.5 (E14.5) (Mebius et al. 2001). Experimental data suggests that LTin cells follow a random walk motion (Veiga-Fernandes et al. 2007) until contact is made with LTo cells residing on the epithelium surface. Contact causes LTo cell differentiation, and the production of factors that promote adhesion of Ltin/LTi cells in the vicinity of the LTo cell (Yoshida et al. 2001). Upon LTi cell contact with a differentiated LTo cell, further adhesion factors are produced, as well as chemoattractants that affect the migration of LTi cells in the vicinity, attracting LTi cells to this growing aggregation of cells (Cupedo et al. 2004; Luther et al. 2003). Cell aggregation continues for a 72-hour period, after which no further cell aggregation is observed (Randall et al. 2008).

It is believed that these interactions lead to the three observable phenomena seen in Fig. 1. The simulation platform we have constructed that models this process can be used to determine how perturbations in modelled biological pathways affect these three observations. For the context of this paper, we are interested in the final observed emergent behaviour in Fig. 1: the development of clusters of LTin and LTi cells (immature PP) at the end of the 72-hour development period. By perturbing parameters that model aspects of the biological system, we can hypothesise the effect a given parameter/biological pathway has on cell aggregation.

## Key model assumptions

In the above description we note that LTin cell migration into the gastrointestinal tract can be detected from E14.5. Flow cytometry data has allowed us to estimate the number of LTin cells in the developing tract 24-hours later, at embryonic day 15.5 (E15.5). The experimental data has been used to estimate that 0.45 % of the gut surface area would be occupied by LTin cells at this point. As we know the surface area of the gut from stereomicroscopy images, and the size of LTin cells, we can estimate the number of cells at E15.5. Cell migration and interactions that lead to PP development are thought to continue for a further 48 h, yet there is currently no experimental data available that specifies cell counts after the E15.5 timepoint. In the absence of this biological data, our model makes two key assumptions that concern the LTin cell population:

### Cell count at E15.5

There is a direct link between the flow cytometry estimate and the number of LTin cells in the model. It has been suggested that LTin cells have a key role in the early initiation of PP development (Patel et al. 2012; Veiga-Fernandes et al. 2007), with LTi cells mainly responsible for cell aggregation at a later stage (van de Pavert and Mebius 2010; Randall et al. 2008) If this hypothesis is true, one could infer that the size of the population of LTin cells in the gut may have an effect on the size and number of PP that develop. To date the effect of a perturbation in the LTin cell population has yet to be established. Our previously published model results (Patel et al. 2012; Alden et al., 2012, 2013) assume that this cell count estimate is an adequate representation of the biological system at that timepoint.

### LTin cell migration rate

Further to the above, in the absence of additional biological data, our model extrapolates the flow cytometry estimate over the 72 h organ development period, producing a linear LTin cell input rate. However, the assumption that cells migrate into the gut in such an ordered manner is questionable. The effect that a perturbation in LTin cell input rate may have on PP development is not currently understood. Our previous explorations utilise the model to examine properties of the cell aggregations that emerge in the gut (immature PP), aggregations that may be affected by this assumption.

For a detailed list of all the assumptions that have been made in the implementation of our model, we direct the reader to our previously published model description (Patel et al. 2012; Alden et al. 2012).

## Method: examining key model assumptions

To further understand the influence of these biological assumptions on predictions made by our PP simulation, we have adopted the following strategies:

### Simulations

To determine the impact of a decrease in the LTin cell population, simulations have been run where the LTin cell number at E15.5 has been calculated from gut surface area percentages ranging from 0.05 to 0.45 % (baseline figure), in 0.05 % increments. To determine any impact of an increase in LTin cell population at E15.5, simulations have been performed that model a 2, 3, 4, and 5 fold increase in LTin cell number. This analysis can be conducted by simply altering parameter values that specify the LTin cell population at E15.5.

Two alternative LTin cell input rates have been explored, replacing the assumed linear migration rate derived from the flow cytometry estimate. The first is an exponential rate where cell migration is initially slow, but increases rapidly. The second utilises a square root function to model the opposite effect: a rapid rate initial rate of migration that then decreases over time. All three LTin cell migration rates lead to the migration of the same number of cells at the E15.5 timepoint. As this is the only timepoint where the number of cells has been estimated from biological data, we deemed it important that this link to the real system remained while the cell migration rate assumption was being explored. To examine this assumption, we have had to implement a new cell migration function within the original simulation, and thus this is more complex than examining the previous assumption detailed above.

As our PP organogenesis simulation is an agent-based implementation, simulated cell behaviour is influenced by pseudo-random number generation. As this has the potential to produce different results for identical parameter conditions, a number of replicate runs are required to ensure the simulation response is representative of the specified parameter conditions. We utilised the consistency analysis technique available in the spartan package (Alden et al. 2013) to determine the minimum number of simulation runs required to mitigate the effect of inherent stochasticity. For each set of conditions, 300 simulation runs were performed. Median values for PP number and area were obtained for each of the 300 runs, producing a distribution of 300 results for each condition examined.

### Statistical analysis

Indication of a change in simulation response is achieved by comparing the distribution of simulation results for a given set of conditions with results from the calibrated baseline, using the Vargha-Delaney A-Test (Vargha and Delaney 2000). The A-Test is a non-parametric effect magnitude test that compares two populations and returns the probability that a randomly selected sample from population one will be larger than a randomly selected sample from population two. Results above 0.71 and below 0.29 indicate a scientifically significant difference between the two populations, with a result of 0.5 indicating no difference. The use of an effect magnitude statistic indicates the extent simulation response changes as the distance between the parameter value and its calibrated value increases.

## Results

### Cell count at E15.5

**Fig. 2:**
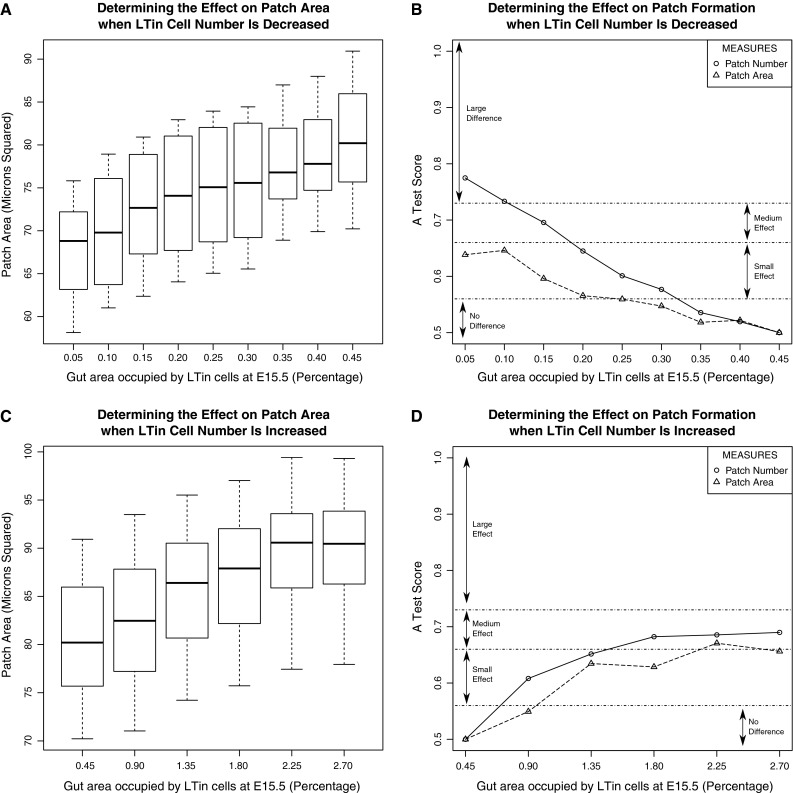
Using our PP organogenesis simulation to assess the assumption that the number of cells in the simulation at E15.5 should match the number observed experimentally. *Top row*: Investigating a decrease in LTin cell number at E15.5. *Upper/lower markers* denote the maximum and minimum value in the distribution respectively. *Bottom row*: Investigating an increase in LTin cell number at E15.5. Simulations were run 300 times for each LTin cell parameter value and median values calculated as described in the method. The *left column* of the figure contains boxplots of the patch area for each value the parameter has been assigned. The *right column* contains the result of a comparison between patch characteristics observed at baseline values and those observed when the parameter is perturbed, using the Vargha-Delaney A-Test (Vargha and Delaney 2000) as described in the method

Figure 2a shows the median PP area obtained for each of the 300 runs, for each experiment where the population size is decreased. It is apparent that the size of PP decreases as the number of LTin cells at E15.5 decreases. Yet cellular aggregations tend to be mainly LTi cells, with LTin cells key in initiating the process (Veiga-Fernandes et al. 2007). For each population size, the simulation median distributions have been compared with the baseline simulation using the Vargha-Delaney A-Test, to establish the size of the effect on simulation response caused by a change in population size (Fig. 2b). It can be noted that the percentage of area from which LTin cell number is calculated can be reduced by 0.10 % before a change in response is observed that the A-Test classifies as ‘small’. Reductions in LTin cell population of greater than 0.25 % lead to a change in patch area response that approaches the effect categorised as ‘medium’ by the A-Test, yet never meets this classification. For patch number however, a clear trend is apparent between the LTin cell population and the number of patches that form in the population. This suggests that the LTin cells may not be a significant factor in cell aggregation, but are key to the number of PP that form in the gastrointestinal tract.

Figure 2c shows the effect of a 2, 3, 4 and 5 fold increase in LTin cell population on PP area. Inversely to the effect above, patch area increases as LTin cell number increases, yet this begins to stabilise after a threefold increase. Figure 2d shows the A-Test scores when the median distributions for each LTin cell population are compared with distributions from the baseline simulation. These data suggest that doubling the number of LTin cells has a small effect on PP area, an effect which increases to ‘medium’ on a threefold increase. However the effect size stabilises after this point, suggesting a further increase in LTin cell number has no effect on the responses observed.

### LTin cell migration rate

**Fig. 3:**
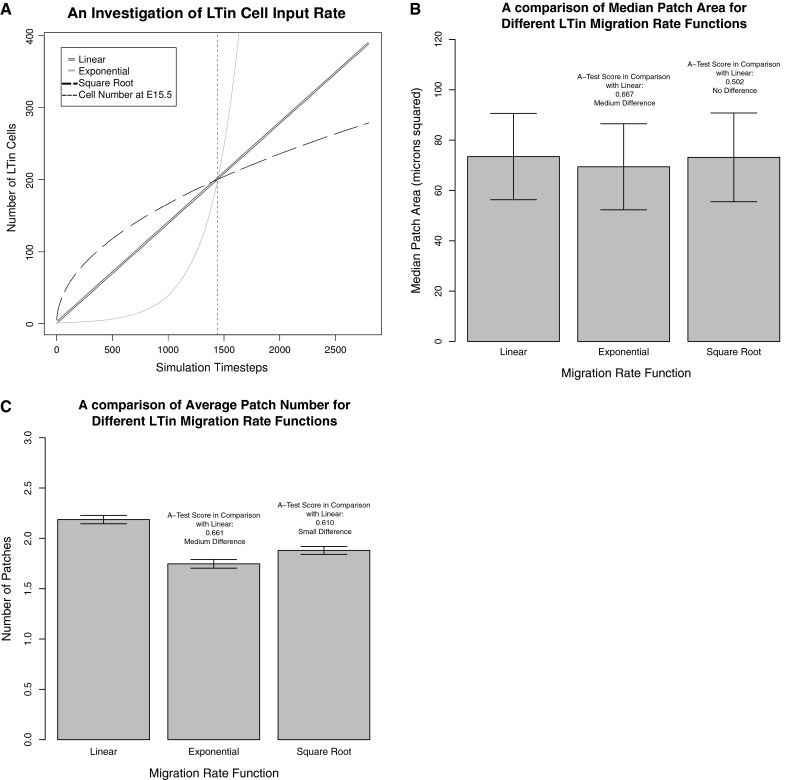
Investigating LTin cell migration rate using our PP organogenesis simulation, by changing the assumed input rate function as described in the Sect. 3. **a** Flow cytometry data has been used to estimate the number of LTin cells present in the gut at E15.5 (*small dotted line*). With cell counts at other timepoints unavailable, the simulator assumes the linear input rate that meets the number of LTin cells observed experimentally, and continues at the same trajectory until E17.5 (*double line*). Alternative migration rates examined here were (i) Exponential (*gray line*) and (ii) Square root (*black broken line*) functions. These three *lines* converge at E15.5 to match the number of cells observed in flow cytometry. 300 simulation runs were performed for each migration rate function and medians calculated as described in the method. **b** A comparison of the median PP area observed for each input rate function. **c** A comparison of the median number of PP for each migration rate function. Results for the exponential and square root functions have been contrasted to the linear input rate using the Vargha-Delaney A-Test (Vargha and Delaney 2000), the result of which is noted on the plot. *Error bars*: Minimum and maximum median patch area

The double line in Fig. 3a shows the LTin cell migration rate throughout the simulation, based on the cell number estimate obtained for E15.5, as explained above. Two alternative rates have been investigated that replace this linear rate: an exponential input rate (grey line) and a square-root function input rate (broken black line). Comparison of the result distributions (Fig. 3b) reveals that a change to a square-root input function, modelling an initial high migration rate that tails off, has no effect on PP area. In terms of average patch number, the A-Test results suggest that the effect size is ‘small’, with slightly fewer patches produced. Replacing the linear input rate with an exponential rate (Fig. 3c) has a ‘medium’ magnitude effect in both PP area and PP size, with fewer, smaller PP produced. This could suggest that initial LTin cell migration could have a key role in controlling PP characteristics (in terms of size and number).

## Discussion

Computer simulations are typically constructed from available biological data and used in the exploration of hypotheses concerning phenomenon that are yet to be fully understood. Our PP organogenesis simulation is a good example of how such an approach can be utilised to produce results that inform wet-lab experimentation. Through adopting the principled design approaches described in the CoSMoS framework (Andrews et al. 2010), the use of biological information in the design of the simulation is clear, as are the areas where biological understanding is incomplete or abstractions are required (Alden et al. 2012). Where abstractions and assumptions were necessary, we have examined each one with experimental biologists and made suitable, well justified implementation decisions. In this paper we have described how such assumptions have been made in the absence of cell counts from multiple time-points in PP development.

Robust simulations of biological processes, usually underpinned by experimental data, are subject to a process of parameter calibration in attempts to replicate a desired emergent behaviour (Read et al. 2012). With replication of behaviour assured, in silico simulations are performed that perturb parameters for which a value is as yet unknown, and statistical techniques utilised to measure the impact of this perturbation (Alden et al. 2013). It is this impact that is commonly reported when demonstrating the use of simulation to explore a biological process.

However, it is rare that simulation developers perform a similar analysis of the assumptions that were necessary in the design of their model. Such an approach would have additional benefits to those gained through parameter perturbation: the assumption could prove to have no impact on simulation behaviour and thus not be an area of interest for experimental biologists, or conversely could indicate that additional biological experimentation is required.

We have examined two key assumptions affecting the migration of LTin cells in the developing gut of the mouse. With cell count data only available for one timepoint, E15.5, this timepoint is a key link between the biological system and the simulation. With the support of biologists specialising in SLO development, we have made assumptions that stem from this data: that the number of cells in the simulation at E15.5 should always match that seen experimentally, and that this figure is reached through a linear migration of LTin cells from E14.5, a migration rate that continues for a further 48 h. In our previous work, we utilised parameter perturbation to explore the effect that simulated biological factors, all of which cannot currently be measured experimentally, have on PP development (Patel et al. 2012; Alden et al. 2012). Here we have taken this further and examined the impact that the decisions made in the design of our simulator (in the *domain* model) have on simulation response.

As described above, the development of PP is highly stochastic, for reasons that are not currently understood (Cornes 1965). In this paper we have undertaken an analysis of the first assumption: that the number of cells at E15.5 matches that observed experimentally. We observe that simulation response is robust when the experimental figure is perturbed by ±20 %, but significant changes in response occur either side of this. The impact of this result is dependent on the strength of the underlying data. If the collaborating biologists are satisfied that the experimental figure is representative across the mouse population, then it would seem that small perturbations would have no significant effect on the end result: the number and size of PP that develop. Conversely, should the number of LTin cells in the mouse gut at this timepoint be over a wider range, the number of cells may impact PP development, explaining some of the variance. This demonstrates why an analysis of implemented assumptions may prove useful: the simulation has identified a window of cell numbers over which the desired behaviour emerges. It is now for the biologists to determine whether this range is biologically plausible. If this is the case, the assumption is fit for our purpose; if not, this would need to be reconsidered.

Our second analysis examines the assumption that LTin cell migration rate is linear: an assumption introduced due to lack of available cell count data from additional timepoints. We observe that a change to a migration rate calculated using a square root function has no impact on simulation response. Viewing this in isolation would suggest that as a change of function would have no impact on response, experimental biologists do not urgently need to seek further cell count data to further understand the biological system. This is a key use of simulation, as this conclusion informs future experimental strategy. Yet a change to an exponential rate does have impact on both simulation responses (PP area and number), suggesting that cell counts may be of interest in future experimental work.

Results such as these demonstrate that a statistical analysis of a perturbation of unknown simulation parameters is not enough when attempting to understand simulation behaviour. Simulation developers need to go further than this and examine how decisions made in the design of their simulation platform impact the simulation response. An analysis such as that detailed above is made much easier with the adoption of a principled approach to simulator design and implementation, such as the CoSMoS framework, where each abstraction and assumption is documented for full scientific scrutiny (Andrews et al. 2010). Collaboration between experimental biologists and developers ensures these design decisions are well justified. Yet this collaboration should then extend to an analysis of these biology-specific decisions, not only for the sake of ensuring a suitable simulation response, but to also ensure that collaborating biologists are aware of the impact such decisions have not only on simulation design but also on the design of future laboratory studies.

## Abbreviations

SLO: Secondary lymphoid organs
PP: Peyer’s patches
CoSMoS: Complex systems modelling and simulation
ABM: Agent-based model
LTi: Lymphoid tissue inducer cell
LTin: Lymphoid tissue initiator cell
LTo: Lymphoid tissue organiser cell
Spartan: Simulation Parameter Analysis R Toolkit Application
